# The extract of an herbal medicine *Chebulae fructus* inhibits hepatocellular carcinoma by suppressing the Apelin/APJ system

**DOI:** 10.3389/fphar.2024.1413463

**Published:** 2024-05-30

**Authors:** Yu-Xi Liu, Lu Wang, Cong-Ying Zhang, Kai-Hua Long, Jing Liu, Shuai Liu, Yuan Wang, Ye Li, Yang Liu, Hong Zhang

**Affiliations:** ^1^ Shaanxi Academy of Traditional Chinese Medicine (Shaanxi Provincial Hospital of Chinese Medicine), Xi’an, China; ^2^ Shaanxi University of Chinese Medicine, Middle Section of Century Avenue, Xianyang, China; ^3^ Basic Medical College, Chifeng University, Chifeng, China

**Keywords:** hepatocellular carcinoma, *Chebulae fructus*, Apelin, APJ, herbal medicine

## Abstract

**Introduction:** Hepatocellular carcinoma (HCC) has been a highly common and pathological disease worldwide, while current therapeutic regimens have limitations. *Chebulae Fructus*, a common herbal medicine in Asia, has been documented to exert potential therapeutic effects on HCC in ancient medicine clinical practice. However, the molecular mechanism underlying its inhibitory effects on HCC requires further investigation.

**Methods:** In this study, the anti-HCC effect of the aqueous extract of *Chebulae Fructus* (CFE) on human HCC and its underlying mechanism were evaluated. Assays including CCK8, EdU staining, crystal violet staining, cell clone formation, flow cytometry, wound healing, and transwell were used *in vitro*. The cell-derived xenograft (CDX) and patient-derived xenograft (PDX) models were used *in vivo*. Transcriptomics analysis, qRT-PCR, ELISA, IHC staining, and Western blotting were employed to determine the mechanism of action of CFE.

**Results:** The results demonstrate that CFE effectively suppressed the proliferation and activity of HepG2 and PLC/PRF/5 HCC cells. CFE also induced apoptosis, and suppressed the migration and invasion abilities of these cells. Furthermore, CFE exhibited inhibitory effects on tumor growth in both H22 and PLC/PRF/5 mouse models, as well as in an HCC PDX model which is derived from patient tumor samples. Moreover, it was identified that CFE treatment specifically suppressed the Apelin/APJ system in HCC cells and tumor tissues. To investigate the role of the Apelin/APJ system in mediating the effects of CFE treatment, an APJ overexpressed cell model is established. Interestingly, it was found that the overexpression of APJ significantly diminished the inhibitory effects of CFE on HCC *in vitro*.

**Discussion:** Collectively, this study provides compelling evidence that CFE exerts significant anti-HCC effects in cell and animal models. Moreover, our findings suggest that the Apelin/APJ system may play a vital role in the therapeutic effects of CFE against HCC.

## 1 Introduction

Global cancer statistics indicate that liver cancer is one of the deadliest forms of cancer worldwide. In 2020, approximately 9,05,700 individuals were diagnosed with liver cancer, leading to a total of 8,32,000 deaths globally (Rumgay et al., 2022). In all types of primary liver cancer, hepatocellular carcinoma (HCC) is the most common one, accounting for 75%–85% of all cases (Ganesan and Kulik, 2023). Current treatments for HCC consist of surgical resection, radiation therapy, chemotherapeutics, and immunotherapy (Daecher et al., 2017; Nam et al., 2020; Mendiratta-Lala, 2021; Tibballs and Clements, 2022; Han et al., 2023). Although surgery remains the primary treatment option for early-stage HCC, its efficacy is unsatisfactory once the disease progresses to the mid- or late-stage. Chemotherapy is not viable for patients with liver dysfunction, and HCC is widely known to be resistant to chemotherapy (Demir et al., 2021). Many patients with advanced HCC receive palliative therapy, which places substantial physical and psychological burdens on them (Vogel et al., 2021). Hence, it is worthwhile to explore innovative and efficient therapies with minimal side effects for managing HCC.

With its extensive clinical background, herbal medicine is increasingly acknowledged as a promising option owing to its distinct advantages, including multiple components, multiple targets, and coordinated intervention effects against HCC (Wang et al., 2020; Lee et al., 2022). Numerous findings indicate that herbal medicine may exert anti-cancer effects through various mechanisms, including modulation of cancer-related signaling pathways, suppression of cell activity, and induction of apoptosis. Over the past decades, pre-clinical and clinical research has been conducted on various herbal medicine extracts and constituents. These studies have demonstrated their significant potential for development into novel anti-HCC agents (Li and Martin, 2011; Chen et al., 2016).

The herb *Terminalia chebula* Retz. is widely allocated in Tibet, Yunnan, and Guangdong provinces of China, and is also extensively used in traditional medicine in Southeast Asian countries like India and Iran (Nigam et al., 2020; Hassan et al., 2022). *Chebulae Fructus*, the mature fruit of *T. chebula* Retz., is utilized to alleviate diarrhea with astringent effects, astringe lung to relieve cough, and reduce inflammation in the throat (Bag et al., 2013; Kim et al., 2022). In ancient medicine, the water decoction of *Chebulae Fructus* was documented as a remedy for liver-related diseases (Bag et al., 2013). Modern medical researches have shown that the aqueous extract of *Chebulae Fructus* (CFE) exhibits notable inhibitory ability on the proliferation of various cancer cells, including osteosarcoma, lung cancer, prostate cancer, and breast cancer cells (Saleem et al., 2002; Shendge et al., 2020). A recent study based on network pharmacology has explored the active constituents and potential mechanisms of CFE in addressing HCC (Jiang et al., 2022). However, further study is required to comprehensively understand the impacts and mechanisms of CFE against HCC.

In the present study, the anti-HCC effects and mechanisms of action of CFE were elucidated using multiple cellular and animal models. The results suggest that CFE exhibits anti-HCC activity by inhibiting the Apelin/APJ system. These findings provide a pharmacological rationale for the application of CFE in the therapy of HCC.

## 2 Materials and methods

### 2.1 CFE preparation

*Chebulae Fructus* (Origin Yunnan, Batch No. 181120) was purchased from Beijing Tong-Ren-Tang company and authenticated by the corresponding author. According to ancient medicinal records, the pericarp of Chebulae Fructus is traditionally used in the decoction form to treat liver-related disorders. Hence, in this study, we removed the seeds from Chebulae Fructus and extracted the pericarp using a decoction pot with double-distilled water (1:10 w/v) at 100°C for 30 min. The supernatant was decanted, and the residue was re-extracted twice using identical conditions, with each cycle for 30 min. The filtered extracts were combined and subsequently concentrated by using the rotary evaporator and the Virtis Freeze Dryer. The final yield of powdered CFE was determined to be 49.82%. The extraction method is referred to previous studies (Sheng et al., 2016).

### 2.2 Cell culture

Human HCC cell lines (HepG2, PLC/PRF/5, Huh7, HCC-LM3, Hep3B) were obtained from Procell Life Science & Technology Co., Ltd. HepG2, PLC/PRF/5, and Hep3B cell lines were cultured in Minimum Essential Medium (MEM; Gibco, United States of America) added with 10% fetal bovine serum (FBS, Gibco) and 1% penicillin-streptomycin (P/S; Invitrogen, United States of America). Huh7 and HCC-LM3 cell lines were cultured in Dulbecco’s Modified Eagle Medium (DMEM; Gibco, United States of America) supplemented with 10% FBS and 1% P/S. Cells were passed when reach 90%–95% confluency in the culture dishes. PLC/PRF/5 cells were separately incubated with GFP-labeled empty lentiviral vector (LV) and human APJ lentivirus (APJ+). The stable APJ + cells (overexpressing APJ) and LV-transfected cells were screened using 1 μg/mL puromycin and then cultured in MEM with 10% FBS, 1% P/S, and 0.5 mg/mL puromycin. All kinds of cells were maintained at 37°C, 5% CO_2_.

### 2.3 Cell viability assays

CCK8 assays were conducted to assess the effects of CFE on viability toward HepG2, PLC/PRF/5, Huh7, HCC-LM3, and Hep3B cells. The cells were treated with various concentrations of CFE or vehicle control (0.1% DMSO) for 24, 48 or 72 h after adhering to the 96-well plates. Following treatment, cell culture medium were removed and the solution of CCK8 was added and incubated for an additional 2 h. Then, absorbance at 450 nm was measured and IC_50_ was calculated. Detection was conducted following a previous methodology (Liu et al., 2023).

### 2.4 EdU (5-ethynyl-2′-deoxyuridine) staining

To detect CFE’s effects on the proliferation of HepG2 and PLC/PRF/5 cells, EdU staining was employed. Cells were treated with CFE (50, 100, 200 μg/mL) or vehicle control (0.1% DMSO) for 48 h. Next, 50 μM EdU was added to the cell culture and incubated for 2 h. Detection was carried out adopting a EdU-647 cell proliferation test kit (Beyotime, China). Fluorescent photos were taken via an inverted fluorescence microscope (Olympus, United States of America). Detection was conducted following a previous methodology (Li et al., 2021).

### 2.5 Cell colony formation

Cell colony formation assays were employed to detect CFE’s effects on cell activity. In brief, cells at a density of 500–1,000 cells/well were seeded in 6-well plates, and treated with CFE (25, 50, 100 μg/mL) or vehicle control (0.1% DMSO) for 14 days. Afterward, cells were incubated with crystal violet (0.1%). Photos were taken via a digital scanner. Detection was conducted following a previous methodology (Li et al., 2021).

### 2.6 Cell apoptosis analysis

Cell apoptosis induced by CFE was assessed adopting Annexin V-FITC/PI staining assays. HepG2 and PLC/PRF/5 cells were incubated with CFE (50, 100, 200 μg/mL) or vehicle control (0.1% DMSO) for 24 h. After the treatment, both suspended and adherent cells were collected and incubated with Annexin V in the dark for 15 min. PI was added before performing the detection. Flow cytometer analysis was then conducted following a previous methodology (Liu et al., 2023).

### 2.7 Cell migration assays

Migratory potential of HepG2 and PLC/PRF/5 cells in response to CFE treatment was evaluated using wound healing assays. When the cultured cells reach 80%–90% confluence, a scratch was made in the middle of each well to create a “wound”. Subsequently, the suspended cells were washed off with phosphate-buffered saline (PBS), and the remain adherent cells were incubated with serum-free MEM containing either CFE (25, 50, 100 μg/mL) or vehicle control (0.1% DMSO). Imaging of the cells was conducted immediately after drug treatment and again after 24 h using a fluorescence microscope. Detection was conducted following a previous methodology (Liu et al., 2023).

### 2.8 Cell invasion assays

For invasion assays, the amount of 1.5 × 10^5^ cells were added to the top chamber with Matrigel-coated membrane of the transwell plate (pore size: 8 μm; BD Biosciences). The MEM used in the top chamber did not contain FBS or growth factors, while the MEM used in the lower chamber was added with 10% FBS to induce the invasion. After incubating the cells with CFE (25, 50, 100 μg/mL) or vehicle control (0.1% DMSO) for 24 h, crystal violet staining was conducted to assess the proportion of invasive cells (Cao et al., 2015).

### 2.9 Establishment of allograft and xenograft mouse models and CFE treatment

The animal experiments conducted in this study were approved by the Ethics Committee of Experimental Animals of Shaanxi Academy of Traditional Chinese Medicine (IACUC No. 2023-10, 2023.11.21). Female Kunming mice, aged 6 weeks, were procured from the medical school of Xi’an Jiao Tong University. H22 mouse HCC cells (5  ×  10^5^ cells) suspended in PBS were subcutaneously implanted into the dorsal area of each mouse. After injection, the mice were evenly separated into 5 groups (n = 6) based on body weight: the vehicle group (PBS), the CFE groups (0.5, 1 or 2 g/kg), and the 5-FU group (positive control, a first-line drug for HCC, 50 mg/kg). The mice were intragastrically (i.g.) treated once daily for 14 days. Throughout the experiment, tumor volume, body weight, clinical signs, and food consumption were monitored. At the end of the experiment, inhalation of 5% isoflurane was conducted to euthanize mice. Tumor of each mouse was stripped and weighed.

Six-week female BALB/c nude mice were obtained from Shanghai Model Organisms Center, Inc. To establish human HCC xenografts, PLC/PRF/5 human HCC cells (5  ×  10^5^ cells) were subcutaneously implanted into the dorsal area of each mouse. Subsequently, mice were divided into 5 groups (n = 6) and treated following the same protocol as the H22 allograft model for 28 days. Upon completion of the treatment, each tumor tissue was collected and separated into several sections for further detection. Immunohistochemistry (IHC) staining and terminal deoxynucleotidyl transferase-mediated dUTP nick end labeling (TUNEL) staining were employed to examine indicators of cell proliferation, cell apoptosis, and angiogenesis within the tumor tissues. Additionally, IHC staining was employed to evaluate APJ expression. Hematoxylin and eosin (H&E) staining was used to analyze whether CFE causes toxicity on main organs of mice. Mouse serum was also collected to perform ELISA assays in the subsequent experiments.

### 2.10 CFE treatment in PDX model

The established female HCC PDX mice were acquired from Shanghai Model Organisms Center, Inc., and divided into 2 groups (n = 7) based on the size of the tumors. Mice in different groups received i. g. administration of either vehicle (PBS) or CFE (1 g/kg) once daily for 14 days. Tumor volume and tumor weight was determined. The animal experiments conducted in this study were approved by the Ethics Committee of Experimental Animals of Shanghai Model Organisms Center (IACUC No. 2020-0047, 2020.12.31).

### 2.11 IHC and H&E assays

The tumor sections were subjected to primary antibodies APJ (#DF4853), Ki-67 (#AF0198), CD31 (#BF8133), or cleaved caspase-3 (#AF7022), which were obtained from Affinity Biosciences Ltd., and then IHC staining was performed. Additionally, the tissue and tumor sections were stained using H&E. Microscopy was utilized to capture the images. The method is referred to a previous study (Liu et al., 2019).

### 2.12 TUNEL staining

TUNEL staining was used to evaluate the apoptosis induced by CFE, vehicle control, and 5-FU in tumors. The tissue sections were pre-treated and detected with the ApopTag Peroxidase *in Situ* Apoptosis Detection Kit (Millipore, CA). The method is referred to a previous study (Liu et al., 2019).

### 2.13 Transcriptome sequencing and analysis

PLC/PRF/5 cells were treated with CFE (100 μg/mL) or vehicle control (0.1% DMSO) for 24 h, and subsequently collected to perform a transcriptome sequencing (Majorbio, Shanghai, China). The RNA-sequence data underwent filtering to identify differentially expressed genes (DEGs). DEGs with |log2FC|≧1 and FDR (Padj) < 0.05 were considered to be with significance. Significant DEGs with a fold change of more than 2-fold were classified to determine their expression patterns under different treatments, to investigate the changes in signaling pathways that influenced by CFE. In addition, functional-enrichment analysis including GO and KEGG were performed to identify which DEGs were significantly enriched in GO terms and metabolic pathways at Bonferroni-corrected *p* < 0.05 compared with the whole-transcriptome background. Genes involved in signal transduction and cancer: overview were identified and their protein-protein interaction networks were constructed. The heatmap displays the top 10 genes.

### 2.14 qRT-PCR analysis

RNA of tumor cells or tissues was extracted using a previously described method (Liu et al., 2019). Subsequently, RT-qPCR analyses were conducted using primers synthesized by Invitrogen (United States). The primer sequences for the genes GAPDH, APJ, Apelin, GNGT1, ADCY10, and PIK3R2 are provided in Table 1. Detection was conducted following a previous methodology (Liu et al., 2023).

**TABLE 1:** Sequences of gene primers.

Gene	Source	Forward (5′-3′)	Reverse (5′-3′)
GAPDH	Human	GGT​GTG​AAC​CAT​GAG​AAG​TAT​GA	GAG​TCC​TTC​CAC​GAT​ACC​AAA​G
APJ	Human	CTC​TGG​ACC​GTG​TTT​CGG​AG	GGT​ACG​TGT​AGG​TAG​CCC​ACA
Apelin	Human	GTC​TCC​TCC​ATA​GAT​TGG​TCT​GC	GGA​ATC​ATC​CAA​ACT​ACA​GCC​AG
GNGT1	Human	GAA​GTG​ACA​CTG​GAA​AGA​ATG​CT	AGG​GAT​TTT​TGT​CCT​CTG​GGA
ADCY10	Human	ACA​AAG​TGT​ACG​ACC​TTC​ATG​C	CGA​AGC​TCA​GAT​AAA​TAG​CCC​TG
PIK3R2	Human	AAA​GGC​GGG​AAC​AAT​AAG​CTG	CAA​CGG​AGC​AGA​AGG​TGA​GTG

### 2.15 Western blotting

Tumor cells or tissues were lysated with RIPA (Liu et al., 2019). The Western blotting assays were conducted with the primary antibodies of APJ (#DF4853) and GAPDH (#AF7021, used as a loading control), which were obtained from Affinity Biosciences LTD. The chemiluminescence imaging method was used to detect protein band signals. The APJ protein signal was normalized. Detection was conducted following a previous methodology (Liu et al., 2023).

### 2.16 ELISA assays

Mouse serum was collected to conduct ELISA assays. The level of Apelin was determined with a commercially available ELISA kit specific to Apelin (#L221107486), following the recommended protocol provided by the manufacturer (Cloud-Clobe Corp., United States). Results were compared with standard curves, and the measurements were performed in triplicate. Detection was conducted following a previous methodology (Liu et al., 2019).

### 2.17 Statistical analysis

Data are shown as mean ± SD. For two-group comparison, the one-way Student T test was performed using the Prism 8.0 software (GraphPad, CA). Statistical significance was set at *p* < 0.05.

## 3 Results

### 3.1 HPLC analysis of CFE

**FIGURE 1:**
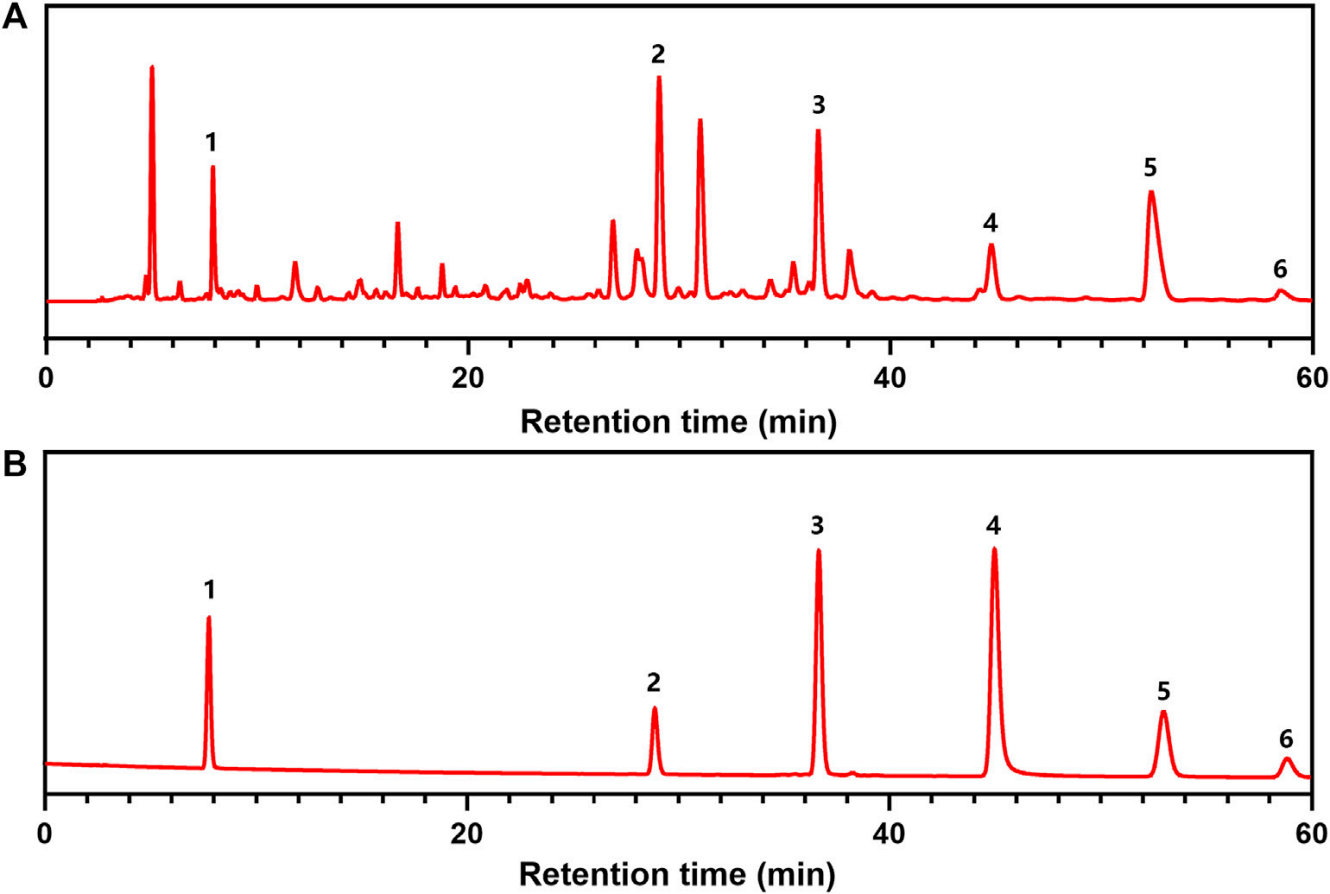
HPLC analyses of CFE. **(A)** HPLC chromatograms of CFE. **(B)** HPLC chromatograms of chemical markers including (**1**) gallic acid, (**2**) corilagin, (**3**) chebulagic acid, (**4**) pentagalloylglucose, (**5**) chebulinic acid, and (**6**) ellagic acid.

HPLC analysis was carried out to examine the components of CFE. Six representative compounds, namely gallic acid, chebulagic acid, pentagalloylglucose, corilagin, chebulinic acid, and ellagic acid, were identified in CFE. HPLC chromatograms of CFE and a mixture containing all six compounds are shown in Figure 1.

### 3.2 CFE inhibits the growth of HCC cells

**FIGURE 2:**
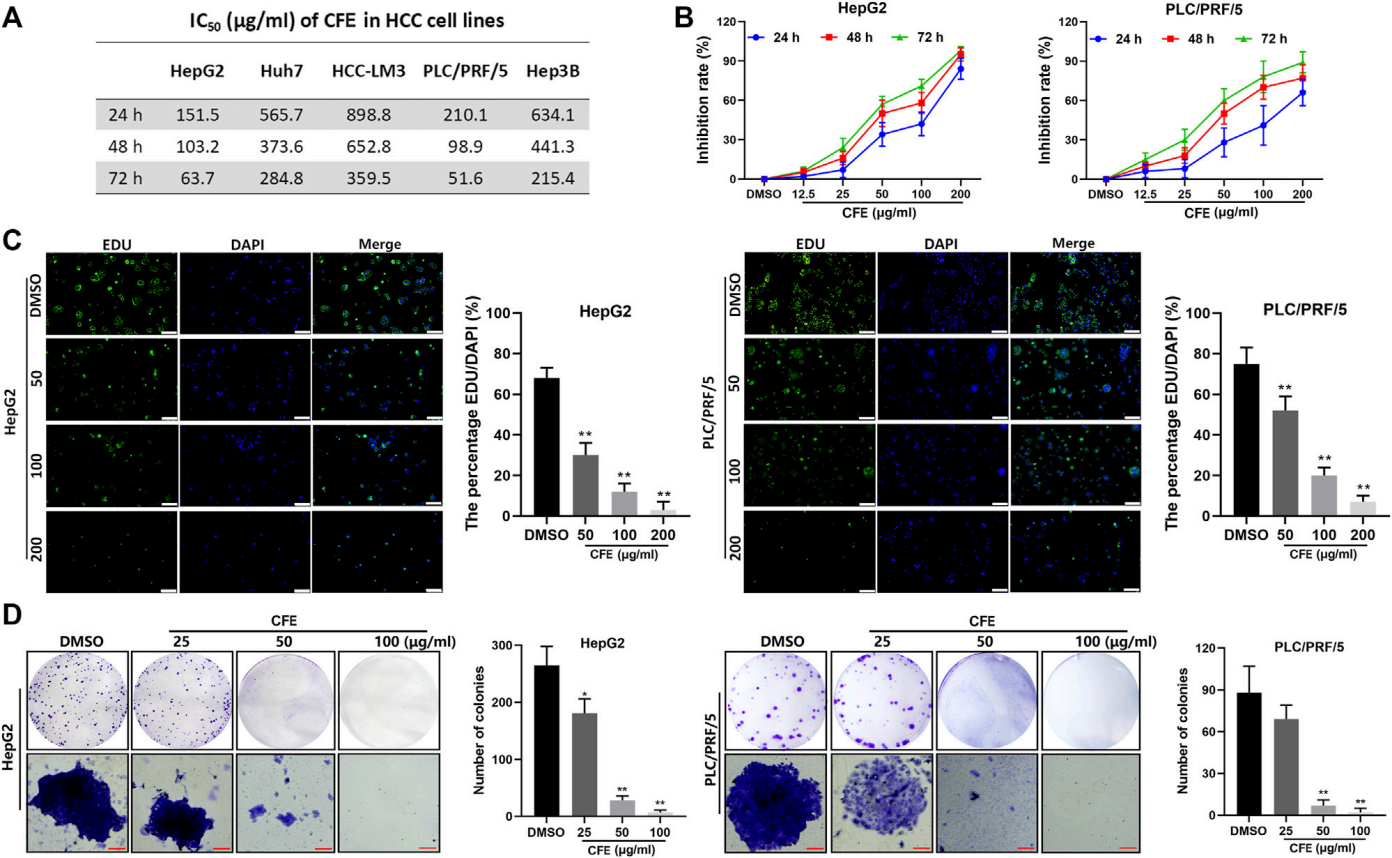
CFE restrains the HCC cell growth. **(A)** IC_50_ of CFE on HepG2, PLC/PRF/5, Huh7, HCC-LM3, and Hep3B cells at 24, 48 and 72 h. **(B)** Dose- and time-dependent effects of CFE on HepG2 and PLC/PRF/5 cells. **(C)** EdU staining of HepG2 and PLC/PRF/5 cells after incubation with the indicated concentrations of CFE or DMSO for 48 h. The representative images captured using an inverted fluorescence microscope are exhibited in the left panels, and the quantification results are presented in the right panels. Scale bar: 100 μm. **(D)** Colony formation ability of HepG2 and PLC/PRF/5 cells treated with the indicated concentrations of CFE or DMSO for 14 days. Quantitative results are shown in the right panels. Scale bar: 100 μm. Data are shown as mean ± SD. *p < 0.05, **p < 0.01.

The IC_50_ of CFE on multiple types of HCC cell lines, including Huh7, HCC-LM3, HepG2, PLC/PRF/5, and Hep3B, was examined and shown in Figure 2A. It was observed that the IC_50_ of CFE in PLC/PRF/5 and HepG2 cells was relatively lower compared to the other cell lines. Hence, these 2 cell lines were chosen to be used in the subsequent experiments. The results of CCK8 assays illustrated that CFE suppressed the proliferation of HepG2 and PLC/PRF/5 cells in a dose- and time-dependent manner (Figure 2B). Additionally, the results of EdU staining assays revealed a marked decrease in the proportion of EdU-positive cells by CFE treatment compared to that by control treatment, indicating a reduction in cell proliferation (Figure 2C). The photos of clone formation assays indicated that the size and amount of clones were obviously reduced in CFE-treated groups compared to the control groups, suggesting that CFE could effectively inhibit cellular activity dose-dependently (Figure 2D). Taken together, these results indicate that CFE can significantly suppress the growth of HCC cells.

### 3.3 CFE induces the apoptosis of HCC cells

**FIGURE 3:**
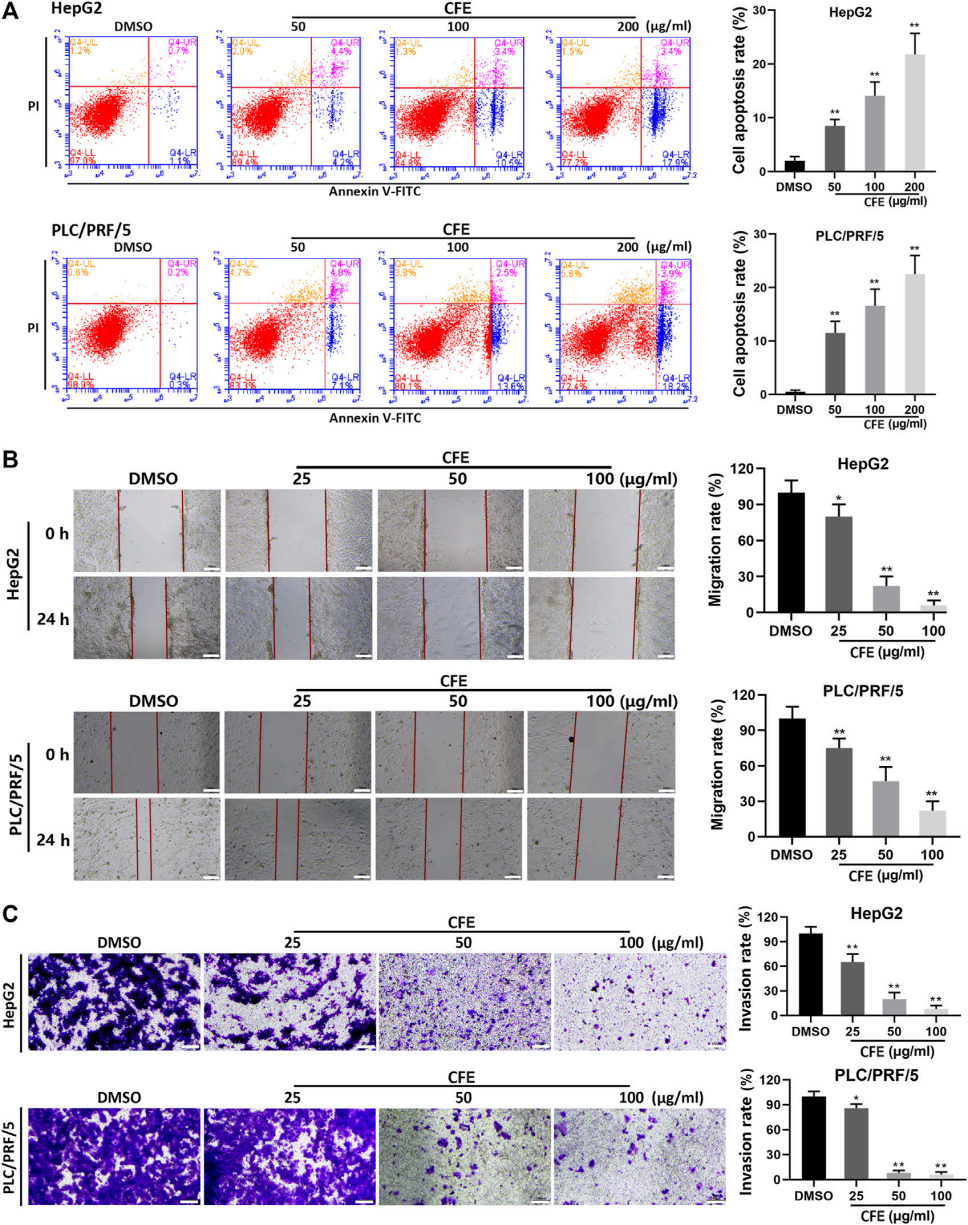
CFE induces HCC cell apoptosis, and inhibits migration and invasion. **(A)** Cell apoptosis of HepG2 and PLC/PRF/5 cells were investigated using flow cytometry after being treated with the indicated concentrations of CFE or DMSO for 24 h. Cell apoptosis rate was determined. **(B)** Wound healing assays in HepG2 and PLC/PRF/5 cells. The wounds before and after CFE treatment for 24 h were shown. Scale bar: 100 μm. **(C)** Transwell assays in HepG2 and PLC/PRF/5 cells treated with CFE for 24 h. The invasive cells were stained with crystal violet and photographed. Quantitative results are shown in the right panels. Scale bar: 100 μm. Data are shown as mean ± SD of three independent experiments. *p < 0.05, **p < 0.01.

The results of Annexin V/PI double staining assays demonstrated that CFE treatment induced apoptosis in HCC cells (Figure 3A). Quantitative analysis revealed that the proportions of apoptotic cells induced by CFE at concentrations of 50, 100, and 200 μg/mL were 8.6% (*p* < 0.01), 13.9% (*p* < 0.01), and 21.3% (*p* < 0.01), respectively, in HepG2 cells. Similarly, CFE at concentrations of 50, 100, and 200 μg/mL resulted in apoptotic rates of 11.9% (*p* < 0.01), 16.1% (*p* < 0.01) and 22.1% (*p* < 0.01), respectively, in PLC/PRF/5 cells.

### 3.4 CFE restrains the migration and invasion of HCC cells

After treated with CFE, significant inhibition of migration was found in both HepG2 and PLC/PRF/5 cells (Figure 3B). The results indicated that CFE at doses of 25, 50, and 100 μg/mL led to a substantial decrease in cell migration by 21.9% (*p* < 0.05), 73.6% (*p* < 0.01), and 91.4% (*p* < 0.01), respectively, in HepG2 cells. Similarly, CFE at doses of 25, 50, and 100 μg/mL resulted in a reduction in cell migration by 26.3% (*p* < 0.01), 50.7% (*p* < 0.01), and 75.5% (*p* < 0.01), in PLC/PRF/5 cells.

The results of transwell assays revealed that treatments with CFE for 24 h significantly suppressed the invasion of HepG2 and PLC/PRF/5 cells (Figure 3C). Specifically, CFE at doses of 25, 50, and 100 μg/mL caused a reduction in cell invasion by 39.5% (*p* < 0.01), 81.9% (*p* < 0.01), and 91.3% (*p* < 0.01) in HepG2 cells, and by 18.2% (*p* < 0.05), 90.8% (*p* < 0.01), and 93.5% (*p* < 0.01) in PLC/PRF/5 cells.

### 3.5 CFE exerts anti-HCC activity in mouse models

**FIGURE 4:**
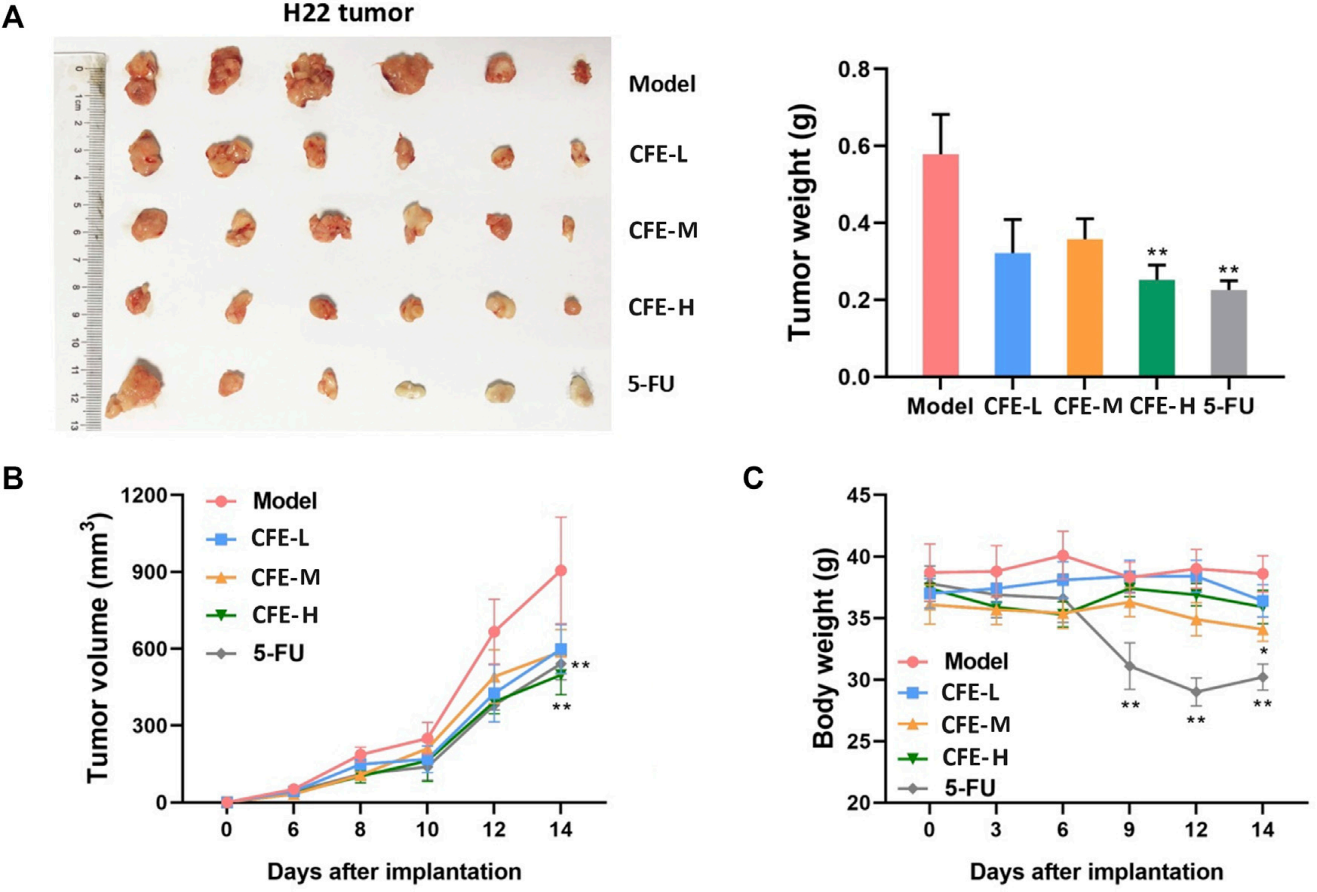
Effects of CFE on H22 tumor growth. **(A)** H22 tumor of different treatment groups, including PBS (Model), low dose of CFE (CFE-L, 0.5 mg/kg), middle dose of CFE (CFE-M, 1 mg/kg), high dose of CFE (CFE-H, 2 mg/kg), and positive control (5-FU). The representative photo of H22 tumors dissected from mice are shown (n = 6) is shown in the left panel, and tumor weight of different treatment groups is shown in the right panel. **(B)** Tumor volume and **(C)** Body weight of H22 tumor-bearing mice during the experiment. *p < 0.05, **p < 0.01.

**FIGURE 5:**
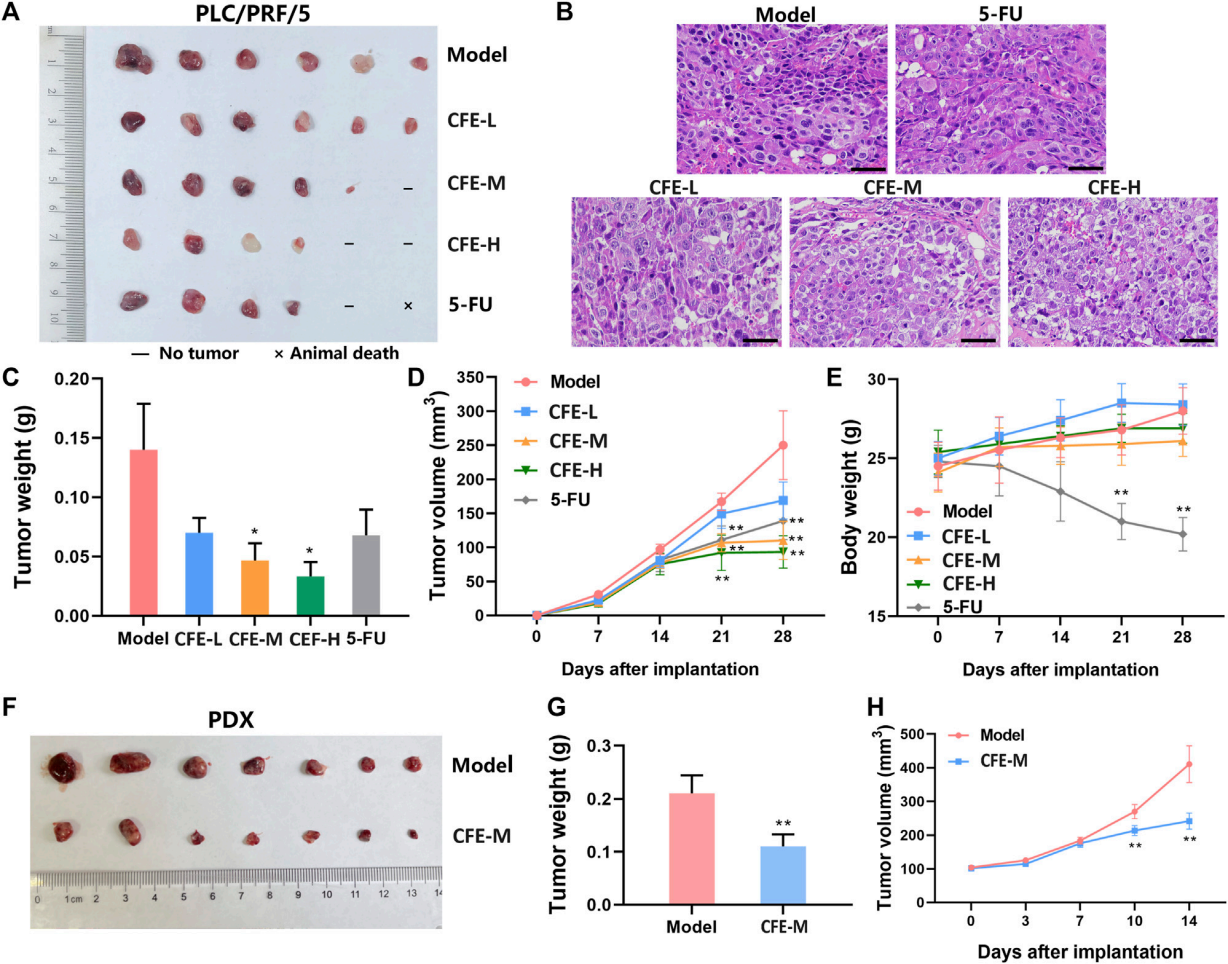
Effects of CFE on PLC/PRF/5 or HCC PDX tumor growth. **(A)** PLC/PRF/5 tumor of different treatment groups, including PBS (Model), low dose of CFE (CFE-L, 0.5 mg/kg), middle dose of CFE (CFE-M, 1 mg/kg), high dose of CFE (CFE-H, 2 mg/kg), and positive control (5-FU). The representative photo of PLC/PRF/5 tumors dissected from mice is shown (n = 6). **(B)** H&E staining of PLC/PRF/5 tumors from different groups. Scale bar: 50 μm. **(C)** PLC/PRF/5 tumor weight of different treatment groups. **(D)** Tumor volume and **(E)** Body weight of PLC/PRF/5-tumor bearing mice during the experiment. **(F)** PDX tumor of PBS (Model) and middle dose of CFE (CFE-M, 1 mg/kg) treated mice. Representative photos of PDX tumors are shown (n = 7). **(G)** PDX tumor weight of different treatment groups. **(H)** PDX tumor volume of mice during the treatment period. All the data were presented as mean ± SD. *p < 0.05, **p < 0.01.

**FIGURE 6:**
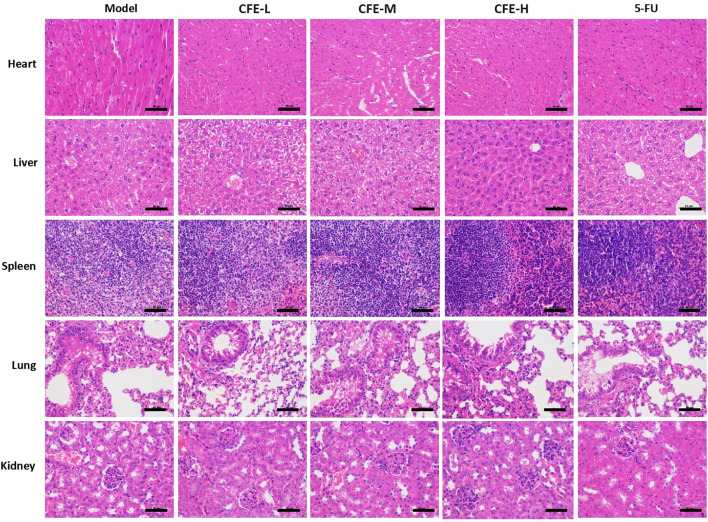
H&E staining of main organs in different PLC/PRF/5 tumor-bearing mice that treated with PBS (Model), low dose of CFE (CFE-L, 0.5 mg/kg), middle dose of CFE (CFE-M, 1 mg/kg), high dose of CFE (CFE-H, 2 mg/kg), and positive control (5-FU). Scale bar: 50 μm.

Allograft, xenograft, and PDX mouse models were employed to assess CFE’s effects on tumor growth. The results demonstrated that the treatment groups receiving CFE exhibited observable decreases in tumor size and weight compared with the control groups in H22 allograft mouse model (Figure 4A,B). Similar results were found in PLC/PRF/5 xenograft (Figure 5A, C, D) and HCC PDX mouse models (Figure 5F–H). Histological examination of tumor tissues indicated that CFE treatment inhibited cell growth in PLC/PRF/5 tumor (Figure 5B). Importantly, CFE did not exhibit notable impact on mouse body weight in both H22 and PLC/PRF/5 tumor-bearing mice. In contrast, 5-FU (positive control, a first-line drug for HCC) treatment resulted in significant weight loss among the mice (Figure 4C; Figure 5E). Furthermore, CFE treatment did not exhibit any noticeable impact on the histopathological features of the main organs in the tested mice (Figure 6). This suggests that the drug is safe for use in mice.

**FIGURE 7:**
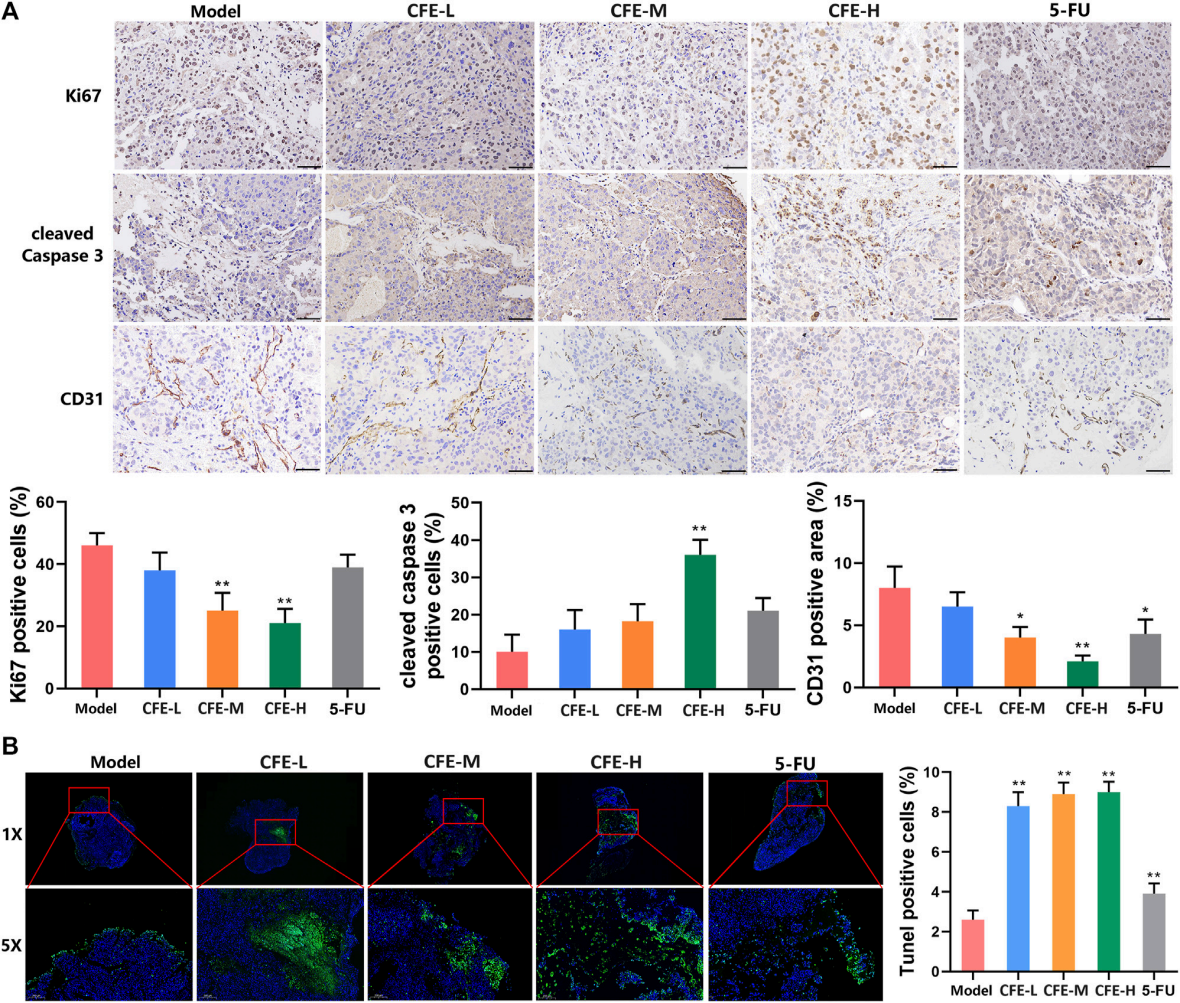
CFE inhibits cell proliferation, causes apoptosis, and suppresses angiogenesis in HCC tumor tissues. **(A)** Ki-67, cleaved-caspase 3, and CD31 in PLC/PRF/5 tumors. Representative IHC staining images are shown in the upper panels. Quantative results are shown in the lower panels. Scale bar: 50 μm. **(B)** TUNEL staining of PLC/PRF/5 tumors. Representative images are shown in the upper panels. The areas in red squares from the corresponding large-scale images (1×) are magnified and shown in the lower images (5×). Quantitative results are presented in the right panels. Data are exhibited as mean ± SD of 3 mice. *p < 0.05, **p < 0.01.

IHC analysis demonstrated that the proportion of the Ki-67-positive cells in tumor tissues of CFE- or 5-FU-treated mice was lower compared with the control group, suggesting that CFE inhibited cell proliferation in tumors (Figure 7A). Moreover, the administration of CFE upregulated the level of an early apoptosis marker cleaved caspase-3 (Figure 7A). This was further supported by TUNEL staining, which demonstrated a more quantity of late apoptotic cells in the CFE groups compared with the control group (Figure 7B). Additionally, results of CD31 (an endothelial maker) staining revealed a decrease in CD31-positive microvessels in the CFE or 5-FU groups compared to the control group, indicating that CFE possesses anti-angiogenesis effects (Figure 7A).

### 3.6 CFE inhibits the Apelin/APJ system

**FIGURE 8:**
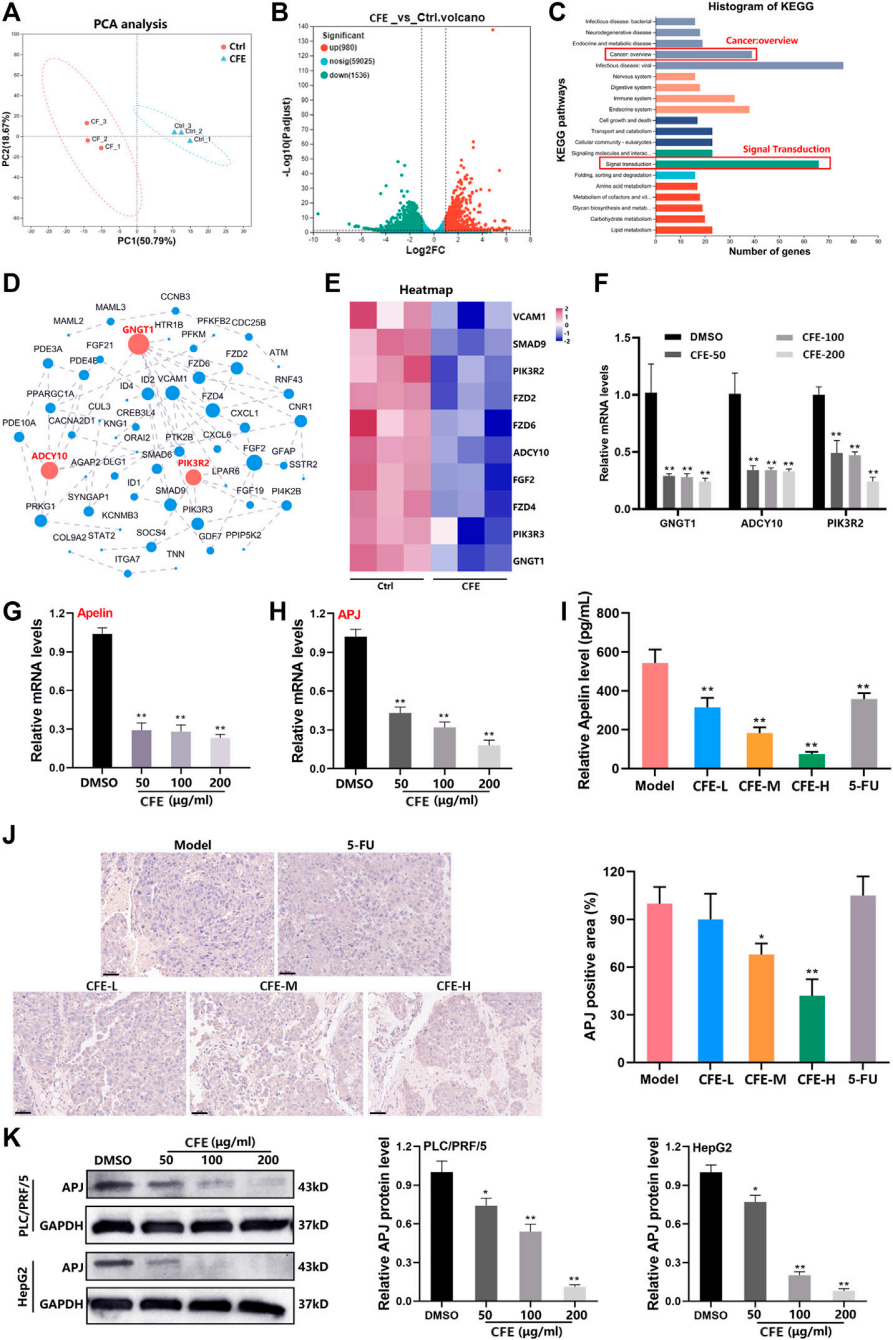
CFE inhibits the Apelin/APJ in HCC cells. **(A)** The transcriptome analysis was performed between ctrl- or CFE-treated PLC/PRF/5 cells. PCA analysis of ctrl and CFE groups. **(B)** The volcano diagram of ctrl and CFE groups. **(C)** The histogram of KEGG pathways. **(D)** Protein-protein interaction network of the differential genes related to signal transduction and cancer: overview. The dot size and number of connections indicate the importance of the protein in the network. **(E)** The heatmap of the top 10 important genes in the protein-protein interaction network. QRT-PCR assays were performed to detect the expression of **(F)** GNGT1, ADCY10, PIK3R2, **(G)** Aplein, and **(H)** APJ in PLC/PRF/5 cells after treated with or without CFE. **(I)** The level of Apelin in the serum of mice treated with vehicle control, CFE, or 5-FU using ELISA assays. **(J)** IHC staining of APJ in tumor tissues from different treatment groups. Scale bar: 50 μm. **(K)** Protein levels of APJ in PLC/PRF/5 and HepG2 cells are determined using Western blotting. GAPDH serves as a loading control. Representative bands are shown in the left panel, and relative band intensities are shown in the right panel. The data were presented as mean ± SD. *p < 0.05, **p < 0.01.

Our data has demonstrated that CFE exhibits anti-HCC activity in both cell and animal models. To further study the changes in gene expression caused by CFE treatment, we treated PLC/PRF/5 cells with 100 μg/mL of CFE for 24 h and performed transcriptome analysis. The principal component analysis (PCA) showed high homogeneity within the two groups (Figure 8A). According to the volcano plot, a total of 980 genes showed significant upregulation, while 1,536 genes exhibited downregulation in response to CFE treatment (Figure 8B). We focused on the CFE-downregulated genes and performed KEGG functional annotation analysis on these genes. The results indicated that the top 3 pathways affected by CFE treatment were related to infectious disease: viral, signal transduction, and cancer: overview (Figure 8C). Considering their relevance to tumors, we chose a total of 85 genes related to signal transduction and cancer: overview for further protein-protein interaction analysis (Figure 8D). The dot size and number of connections represent the importance of the proteins in the network. Hierarchical clustering analysis was performed on the top 10 genes with the highest relevance (Figure 8E). To verify these findings, we detected the mRNA levels of the top 3 important genes: GNGT1, ADCY10, and PIK3R2 in PLC/PRF/5 CFE-treated or -untreated cells. Consistently, CFE downregulated the mRNA expression level of these genes (Figure 8F). Through analysis of the KEGG pathway database, we found that GNGT1, ADCY10, and PIK3R2 are associated with a common pathway known as the Apelin/APJ system. CFE inhibited the mRNA levels of Apelin and APJ in PLC/PRF/5 cells (Figure 8G, H). Results of ELISA assays showed that CFE decreased the level of Apelin in mouse serum (Figure 8I). IHC staining of APJ indicated that CFE suppressed the expression of APJ in tumor tissues (Figure 8J). Furthermore, results of Western blotting demonstrated that CFE downregulated the protein level of APJ in PLC/PRF/5 and HepG2 cells dose-dependently (Figure 8K). Overall, these findings demonstrate that CFE can significantly inhibit the Apelin/APJ system in HCC cells and tumor tissues.

### 3.7 Overexpression of APJ attenuates CFE’s anti-HCC effects

**FIGURE 9:**
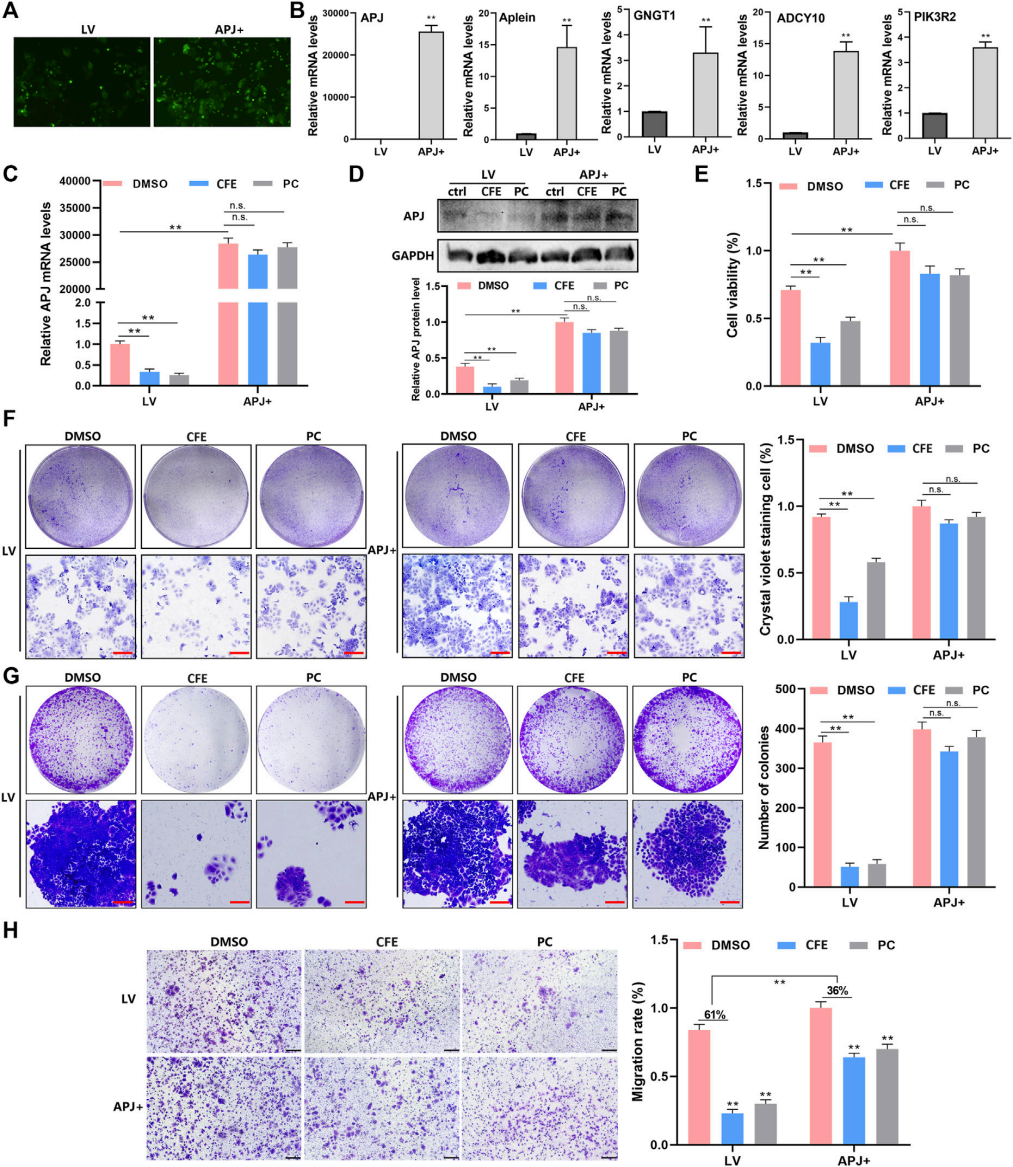
Overexpression of APJ diminishes the effects of CFE on HCC cells. **(A)** PLC/PRF/5 cells were separately incubated with GFP-labeled empty lentiviral vector (LV) and human APJ lentiviral (APJ+). The transfection is detected using the inverted fluorescence microscope. **(B)** QRT-PCR assays were performed to detect the expression of APJ, Apelin, GNGT1, ADCY10, and PIK3R2 in APJ + cells and LV-transfected cells. **(C)** APJ expression in APJ + cells and LV-transfected cells treated with DMSO, CFE (100 μg/mL), or positive control (PC, APJ inhibitor, ML221, 10 μM). **(D)** Protein levels of APJ in APJ + cells and LV-transfected cells treated with DMSO, CFE, or positive control. **(E)** CCK8 assays in APJ + cells and LV-transfected cells treated with DMSO, CFE, or positive control. **(F)** Crystal violet staining of APJ + cells and LV-transfected cells treated with DMSO, CFE, or positive control. Scale bar: 100 μm. **(G)** Cell colony formation assays of APJ + cells and LV-transfected cells treated with DMSO, CFE, or positive control. Scale bar: 100 μm. **(H)** Transwell assays of APJ + cells and LV-transfected cells treated with DMSO, CFE, or positive control. Scale bar: 50 μm. Results are presented as mean ± SD. *p < 0.05, **p < 0.01.

To investigate the function of APJ in the suppressive effects of CFE on HCC, we established stable cells that overexpressing APJ. PLC/PRF/5 cells were incubated separately with a GFP-labeled empty lentiviral vector (LV) and human APJ lentivirus (APJ+), and the stable cells were selected by puromycin (Figure 9A). APJ, Apelin, GNGT1, ADCY10, and PIK3R2 were found to have elevated mRNA levels in cells overexpressing APJ compared with empty vector-transfected cells (Figure 9B). Next, the effects of CFE on APJ levels in APJ + cells and LV-transfected cells were exmained. QRT-PCR and Western blotting expounded that overexpressing APJ reduced the suppressive effects of CFE on APJ mRNA and protein levels (Figure 9C, D). Furthermore, we investigated CFE’s effects on viability, growth, activity, and invasion in APJ + cells and LV-transfected cells. CCK8 assays and crystal violet staining showed that overexpression of APJ diminished CFE’s effects on HCC cell proliferation and growth (Figure 9E, F). Cell clone formation assays showed that APJ overexpression reduced CFE’s inhibitory effects on HCC cell activity (Figure 9G). Transwell assays indicated that APJ overexpression attenuated CFE’s effects on HCC cell invasion (Figure 9H). In conclusion, these findings highlight that the inhibition of APJ contributes to the mechanisms through which CFE exerts its anti-HCC effects.

## 4 Discussion

Despite the development of various treatment methods, liver cancer seriously threatens human health (Gravitz, 2014). Unfortunately, liver cancer is often diagnosed in the advanced stage due to the lack of typical symptoms during the early stages (Pinero et al., 2020). By the time it reaches the advanced stage, the tumor has already migrated to other parts of the body or causes severe liver dysfunction, which poses a great challenge to treatment (Anwanwan et al., 2020). Recently, herbal medicine has shown significant promise in the management of various cancers (Hu et al., 2016; Zhong et al., 2019). The advantage of herbal medicine lies in its ability to enhance immune function and improve the life quality of patients, with minimal side effects and low toxicity (Ying et al., 2018; Suroowan and Mahomoodally, 2019).

**FIGURE 10:**
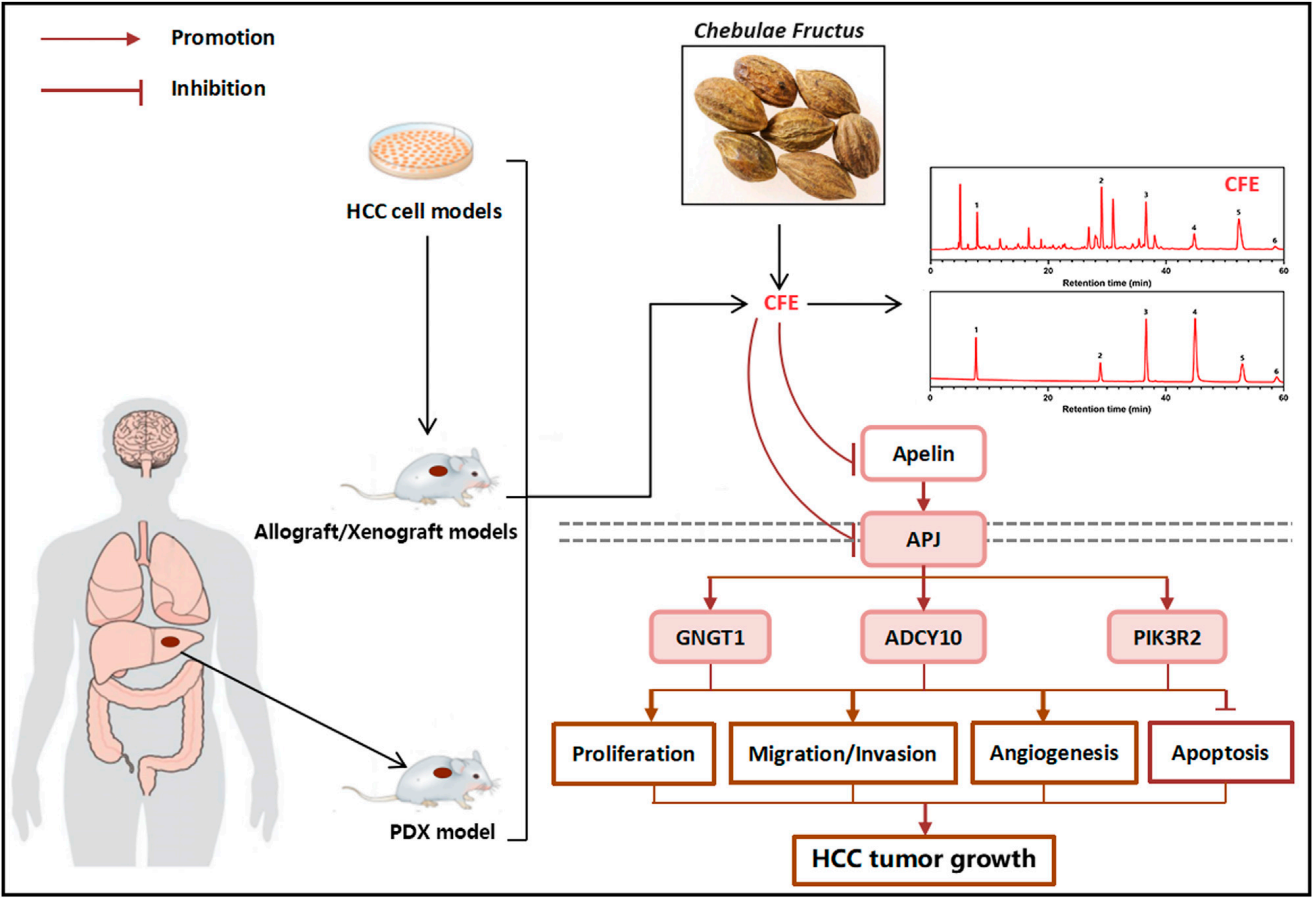
Schematic diagram of the effect and mechanism study of CFE on HCC.

Both traditional and modern research have demonstrated the potential of the herbal medicine *Chebulae Fructus* in treating HCC (Bag et al., 2013; Jiang et al., 2022). Nevertheless, the impacts and mechanisms by which CFE affects HCC have yet to be fully comprehended. The intention of this study was to elucidate the anti-tumor activity of CFE in both HCC cellular and mouse models. Initially, we conducted *in vitro* experiments to investigate the impact of CFE on cell proliferation, growth, apoptosis, migration, and invasion. Through the CCK8 and EdU assays, it was observed that CFE effectively inhibited proliferation and growth of HCC cells dose- and time-dependently. Additionally, colony formation assays indicated suppressed cell activity upon treatment with CFE. Moreover, flow cytometry analysis revealed that CFE induced cell apoptosis dose-dependently. Furthermore, the results of wound healing and transwell assays exhibited that CFE reduced HCC cell migration and invasion dose-dependently. These results collectively suggest that CFE shows anti-tumor activity in HCC cell models. Subsequently, *in vivo* experiments were conducted using mouse models to assess the anti-HCC effects of CFE. The mice were separated into a control group, a positive control group, and groups treated with various doses of CFE. Throughout the study, the various parameters of tumor growth, such as volume and weight were measured. The results clarified that treatment with CFE obviously decreased tumor volume and weight both in the H22 and PLC/PRF/5 tumor-bearing mouse models. Similar results were found in the PDX mouse model. Furthermore, IHC staining was carried out to examine markers of cell proliferation, cell apoptosis, and angiogenesis within the PLC/PRF/5 tumor tissues. TUNEL staining was also conducted. The results indicated that CFE suppressed cell proliferation, caused cell apoptosis, and restrained angiogenesis *in vivo*. These findings offered significant understanding of how CFE impacts the growth and advancement of tumors. More importantly, during the administration process, the CFE-treated group did not show observable change in mouse body weight, whereas the 5-FU-treated group showed a noticeable decrease in body weight. Additionally, the results of H&E staining exhibited that after 28 days of treatment, CFE did not cause apparent damage to main organs of mice. These findings indicate that CFE has lower toxicity compared to 5-FU, and should be safe for use in mice. To investigate the mechanism of action of CFE in inhibiting HCC, transcriptome analysis was conducted. Ten genes were screened by KEGG functional annotation analysis and protein-protein interaction analysis. The results were subsequently validated using qRT-PCR. Notably, CFE significantly downregulated the mRNA levels of the top 3 genes: GNGT1, ADCY10, and PIK3R2. Further analysis using the KEGG pathway database revealed that GNGT1, ADCY10, and PIK3R2 share a common upstream pathway known as Apelin/APJ. Apelin expression is increased in different types of cancer, and the Apelin/APJ system plays a crucial role in tumor development by promoting cell proliferation, angiogenesis, metastasis, cancer stemness and drug resistance. In addition, Apelin can inhibit the apoptosis of cancer cells (Masoumi et al., 2020). The Apelin/APJ system has also been recognized to be closely associated with the development of HCC. Using a small molecule inhibitor of Apelin/APJ effectively inhibits the Apelin-PI3K/Akt signaling pathway and HCC growth *in vitro* and *in vivo* (Chen et al., 2019). Furthermore, it has been observed that Apelin/APJ is highly expressed in HCC and plays a vital role in promoting arteriogenesis in the tumor (Muto et al., 2014; Lee et al., 2019). This suggests that Apelin/APJ may be a promising target for the treatment of HCC. In the current study, results of qRT-PCR, ELISA, IHC staining, and Western blotting collectively demonstrated that CFE could significantly inhibit the Apelin/APJ system in HCC cells and tumors. To understand the role of APJ in the anti-HCC effects of CFE, the stable cells that overexpressing APJ were established. Results of qRT-PCR and Western blotting illustrated that overexpressing APJ attenuated the inhibitory effects of CFE on APJ mRNA and protein levels. Moreover, overexpressing APJ diminished CFE’s suppressive effects on HCC cell proliferation, growth, activity, and invasion. Overall, this study provides evidence for the anti-HCC effects of CFE and elucidates the mechanism involving the Apelin/APJ system. These results suggest that CFE could be developed into a therapeutic agent for treating HCC with low toxicity (Figure 10).

A major challenge in modern herbal medicine research is the unclear composition of herbal remedies (Wu et al., 2013; Yan et al., 2014). Previous studies have utilized various analytical techniques such as HPLC, UPLC, or LC-MS to analyze the chemical components present in Chebulae Fructus (Sheng et al., 2016; Li et al., 2022; Zhao et al., 2022). In this study, we have characterized the components of CFE and identified its characteristic constituents, including gallic acid, corilagin, chebulagic acid, pentagalloylglucose, chebulinic acid, and ellagic acid using HPLC. For future studies, we intend to develop a fingerprint of CFE and optimize its extraction process.

## 5 Conclusion

To make a conclusion, our data has clarified that CFE exhibits suppressive effects against HCC both in cellular and animal models, and inhibiting the Apelin/APJ system contributes to the anti-HCC mechanisms of CFE. These findings provide evidence to support the development of CFE as a contemporary therapeutic agent for the management of HCC, and further validate the traditional use of CFE in managing liver-related ailments. Additionally, this study indicates that targeting the Apelin/APJ system holds significant potential as a promising strategy against HCC.

## Data Availability

The data presented in the study are deposited in the NCBI repository, accession number PRJNA1110043. This is available at: https://www.ncbi.nlm.nih.gov/search/all/?term=PRJNA1110043.

## Abbreviations

HCC, hepatocellular carcinoma; CFE, the aqueous extract of *Chebulae Fructus*; PDX, patient-derived xenograft; MEM, Minimum Essential Medium; DMEM, Dulbecco’s Modified Eagle Medium; FBS, fetal bovine serum; P/S, penicillin-streptomycin; LV, lentiviral vector; APJ+, APJ lentivirus; PBS, phosphate-buffered saline; i.g., intragastrically; IHC, immunohistochemistry; TUNEL, terminal deoxynucleotidyl transferase-mediated; dUTP, nick end labeling; H&E, hematoxylin and eosin.
