# Supplementary figures and images for: The extract of an herbal medicine Chebulae fructus inhibits hepatocellular carcinoma by suppressing the Apelin/APJ system
Source: Front Pharmacol. 2024 May 30;15:1413463. doi: 10.3389/fphar.2024.1413463 (PMC11177762; doi:10.3389/fphar.2024.1413463)

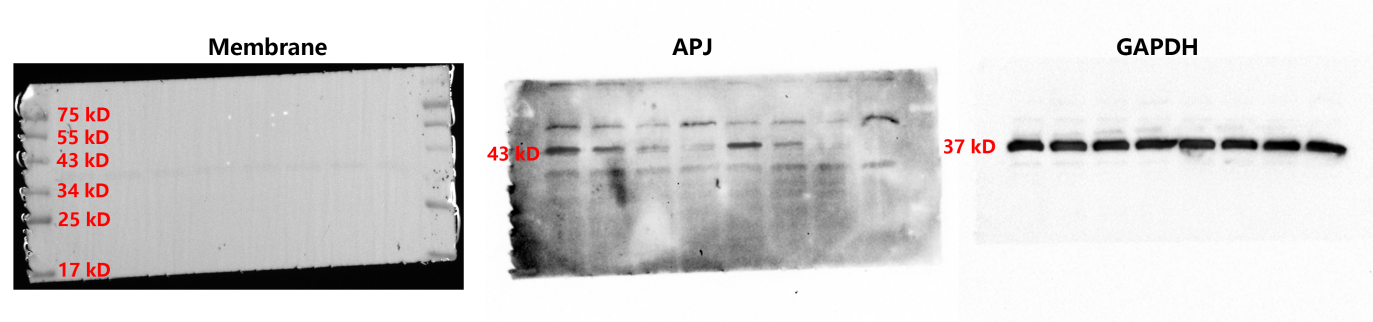


The original blots of APJ and GAPDH in Figure. 8K.


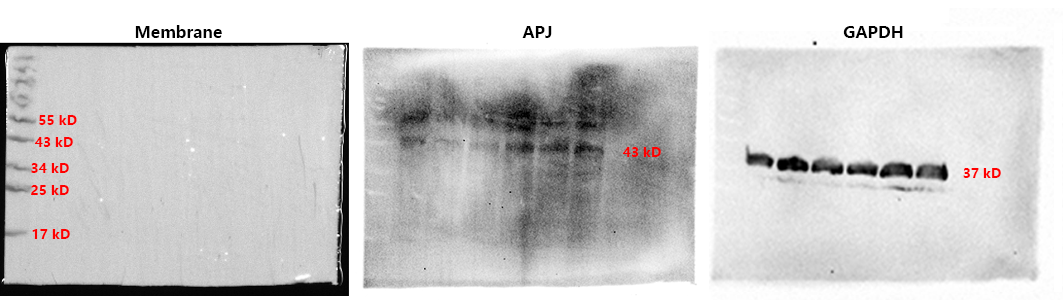
The original blots of APJ and GAPDH in Figure. 9D.

Supplement: Supplementary file 1 [file DataSheet1.docx]
